# High-Accuracy Gaze Estimation for Interpolation-Based Eye-Tracking Methods

**DOI:** 10.3390/vision5030041

**Published:** 2021-09-15

**Authors:** Fabricio Batista Narcizo, Fernando Eustáquio Dantas dos Santos, Dan Witzner Hansen

**Affiliations:** 1 Eye Information Laboratory, Department of Computer Science, IT University of Copenhagen (ITU), 2300 Copenhagen, Denmark; witzner@itu.dk; 2 Office of CTO, GN Audio A/S (Jabra), 2750 Ballerup, Denmark; 3 Federal Institute of Education, Science and Technology of the Northern Minas Gerais (IFNMG), Diamantina 39100-000, MG, Brazil; fernando.dantas@ifnmg.edu.br

**Keywords:** high-accuracy gaze estimation, uncalibrated setup, gaze-mapping calibration, eye-tracking, eye tracker

## Abstract

This study investigates the influence of the eye-camera location associated with the accuracy and precision of interpolation-based eye-tracking methods. Several factors can negatively influence gaze estimation methods when building a commercial or off-the-shelf eye tracker device, including the eye-camera location in uncalibrated setups. Our experiments show that the eye-camera location combined with the non-coplanarity of the eye plane deforms the eye feature distribution when the eye-camera is far from the eye’s optical axis. This paper proposes geometric transformation methods to reshape the eye feature distribution based on the virtual alignment of the eye-camera in the center of the eye’s optical axis. The data analysis uses eye-tracking data from a simulated environment and an experiment with 83 volunteer participants (55 males and 28 females). We evaluate the improvements achieved with the proposed methods using Gaussian analysis, which defines a range for high-accuracy gaze estimation between $-0.5^{\circ }$ and $0.5^{\circ }$. Compared to traditional polynomial-based and homography-based gaze estimation methods, the proposed methods increase the number of gaze estimations in the high-accuracy range.

## 1. Introduction

Researchers and companies constantly aim to improve eye trackers’ accuracy and precision. Accuracy is the average difference between the gaze estimation and the actual stimuli position. On the other hand, precision is the eye-tracking method’s reliability to reproduce the same gaze estimation in successive samples. This work refers to the mapping from gaze estimation onto ground truth as gaze error in pixels or visual angle degrees. Some gaze estimation methods can achieve high-accuracy when the gaze error is $0.5^{\circ }$ or less. High-accuracy gaze estimation is essential to describe the actual user’s Point-of-Regard (PoR) truthfully. Some applications with minimal stimulus require very accurate gaze estimation, such as reading analysis, attention maps, human–computer interaction, among others, and small uncertainties could be very critical to such studies.

In general, video-based eye-tracking methods extract features from the eye image (e.g., pupil center, iris center, eye corners, eyeball center, glints) to map coordinates from the user’s eyes plane to coordinates in a viewed plane. The viewed plane in remote eye trackers (RET) usually is a computer monitor, and in head-mounted eye trackers (HMET) usually is an image from a scene camera to represent the user’s field-of-view. There are two types of feature-based eye-tracking [1] methods, namely: (1) interpolation-based, which uses polynomial regression or projective geometry to estimate the PoR in a 2D plane; and (2) model-based, which uses the eye feature to create a tridimensional geometric model of the eye and estimate the PoR in the 3D space.

Interpolation-based methods and off-the-shelf eye trackers are the most commonly used technologies in academic studies because they are easier to implement than model-based methods. Interpolation-based methods require an individual gaze-mapping calibration to adjust unknown coefficients of the gaze estimation method. During the gaze-mapping calibration, the user looks at a set of calibration targets, while the eye-tracking system collects the corresponding eye feature coordinates. After fitting the corresponding points from the eye plane and viewed plane, the eye-tracking system is able to estimate the user’s gaze on the entire viewed plane. It is necessary to perform the gaze-mapping calibration before starting a new eye-tracking session to achieve precise gaze estimations, especially in uncalibrated setups. However, for general use, some fully calibrated setups require only a single gaze-mapping calibration per user.

Despite the high-accuracy gaze estimation achieved just after the gaze-mapping calibration, interpolation-based methods usually decrease their accuracy because they are susceptible to various factors, such as low-resolution eye images [2,3], natural head movements [4,5], poor gaze-mapping calibration [6,7], eye occlusions [8,9], the geometry of eye tracker components [10,11], nonlinearity of eye feature distribution [4,12], among others [1,13]. The eye-camera location has an essential role in the gaze estimation accuracy in both RET and HMET because the location defines the perspective and distribution of the eye feature on the eye image plane. The gaze error changes according to the relative position between the viewed plane and the eye tracker device, and between the eye-camera and the user’s eye.

To address these limitations, we propose a set of geometric transformation methods to reduce the eye-camera location’s negative influence in interpolation-based eye-tracking methods. The proposed methods only require the traditional information available in the gaze-mapping calibration (i.e., eye feature and targets), and they are suitable for uncalibrated, partially and fully calibrated setups. We exploit the crucial observation that creates a virtual perspective camera aligned with the *x*- and *y*-axes of the user’s eyes would capture a uniform eye feature distribution independently of the eye-camera location. We thus design an experiment using simulated and real eye-tracking data to assess the influence of different camera locations and radial distortion in the eye feature distribution.

This work describes the effectiveness of the proposed geometric transformation methods based on eye-camera realignment and eye feature distribution undistortion to achieve higher accuracy than traditional interpolation-based eye-tracking methods. The contributions of our work are summarized as follows:

- A novel method to compensate for the influence of eye-camera location in gaze estimation based on virtual perspective camera alignment (Section 2.1). Contrary to traditional interpolation-based methods, the proposed method uses a normalized plane between the eye plane and the viewed plane to align the eye-camera in the center of the optical axis, and thus gains unrestricted eye-camera placement for uncalibrated and fully calibrated eye trackers.
- A novel method to undistort eye feature distribution on the eye plane (Section 2.2). After aligning the eye-camera onto the optical axis, the eye feature distribution will be symmetric and uniform centered in the eye feature distribution. However, due to the nonlinear projection of eyeball on the eye plane, the eye feature distribution presents a radial distortion. This method uses the distortion coefficients to reshape the eye feature distribution in an almost linear dispersion.
- This work introduces a new open-source dataset for eye-tracking studies called EyeInfo dataset (available on https://github.com/fabricionarcizo/eyeinfo, accessed on 17 August 2020). This dataset contains high-speed monocular eye-tracking data from an off-the-shelf remote eye tracker using active illumination. The data from each user has a text file with annotations concerning the eye feature, environment, viewed targets, and facial features. This dataset follows the basic principles of the General Data Protection Regulation (GDPR).

The remaining of the paper is organized as follows: Section 2 introduces the problem formulation of the eye-camera location’s influence and describes the mathematical developments and the proposed compensation method. Section 2 also presents nonlinear eye feature distribution issues and derives the learning algorithm from reducing the radial distortion. Section 3 describes the experiments with real and simulated eye-tracking data and demonstrates promising results, Section 4 discusses some further aspects of the proposed methods, and Section 5 concludes the paper.

## 2. Materials and Methods

This section reflects on the eye-camera location’s influence on interpolation-based eye-tracking methods. It also provides information about the methodology applied to compensate for the eye-camera location and improve gaze estimation accuracy. Therefore, this section proposes two distinct methods, i.e., eye-camera location compensation and eye feature distribution undistortion. The former focuses on figure out how the gaze estimation accuracy changes according to the eye-camera location; the latter underlines the problems relate to the non-coplanarity eye plane in interpolation-based methods. Appendix A presents a summary of the most popular methods to estimate the user’s gaze, and Hansen and Ji [1] present a more detailed overview of eye-tracking models.

### 2.1. Eye-Camera Location Compensation Method

This subsection proposes a method to compensate for the eye-camera location’s influence on gaze estimation accuracy. It first uses the eye-tracking data (e.g., pupil centers) from the gaze-mapping calibration to create a normalized space, between the eye plane and the viewed plane, for the eye feature distribution. The correlation between a normalized eye feature and its corresponding viewed calibration target is similar to physically aligning the eye-camera close to the eye’s optical axis. Therefore, the proposed method uses the normalized eye feature, without the influence of the eye-camera location, as the input data for the eye-tracking pipeline.

The eye-camera captures images from the user’s eyes in video-based eye trackers, aiming to monitor eye information essential to the eye-tracking system. In general, monocular eye-tracking systems use one eye-camera to monitor a single eye activities [14,15,16]. However, there are also binocular eye-tracking systems that can use one eye-camera to monitor both eyes simultaneously [17,18,19], multiple synchronized cameras to monitor each eye individually [20,21], or multiple eye-cameras to capture images from the same eye in different perspectives for 3D reconstruction [2,22,23].

Eye trackers place the eye-cameras to optimize the capture of high-quality eye images and avoid blocking the user’s field of view and the viewed plane. The eye-camera location must support the eye-tracking system to monitor even large eye movements and provide the main tracked eye feature during the entire eye-tracking session. In general, RET places the eye-camera under the computer screen in a range of 50–60 cm from the user. On the other hand, HMET places the eye-camera close to the user’s eye at a considerable angle between the eye-camera and the eye’s optical axis.

**Figure 1:**
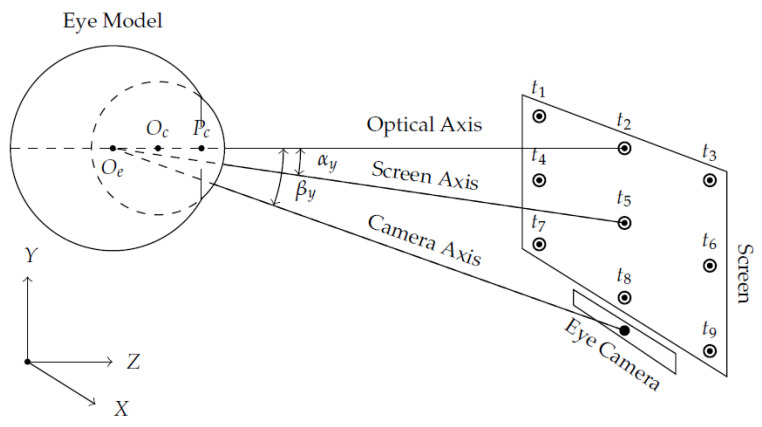
This geometric relationship shows schematic representations of the eye, eye-camera, and screen in a remote setup. Gullstrand–Le Grand Eye Model represents a simplified mathematical model for the human eye as (i) a set of two spheres with distinct size to describe the eyeball, and corneal surface; (ii) the rotation of the eye around a fixed point ($O_{e}$); and (iii) the optical axis that passes through the eyeball center ($O_{e}$), cornea center ($O_{c}$), and pupil center ($P_{c}$), and coincides with the calibration target $t_{2}$. The line that joins the eyeball center and the center of the screen corresponds to the screen axis. The eye-camera is under the screen and aligned horizontally with the center of the screen, and its axis joins the eyeball center and the camera center.

Figure 1 illustrates the geometric relationship between the user’s eye, eye-camera, and computer screen in a remote setup. The relative geometry of the components defines two central angles, namely: (1) α angle between the optical axis and screen/scene axis, and (2) β angle between the optical axis and eye-camera axis. Due to eye trackers’ geometry, the tracked eye feature distribution changes according to α and β angles. We hypothesize that changes in the shape and coordinates of the eye feature distribution could substantially impact interpolation-based eye-tracking methods. Therefore, it is crucial to understand the geometry and the locations of some eye tracker components to reduce the influence of large α and β angles into the gaze estimation.

**Figure 2:**
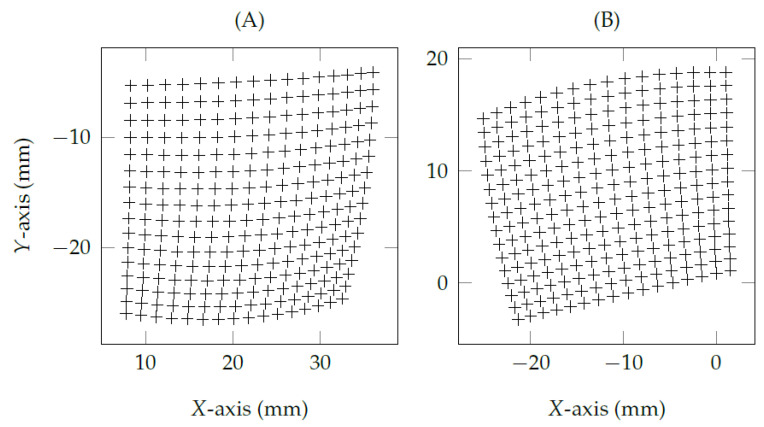
The eye-camera location changes the shape and coordinates of a nonlinear eye feature distribution. The crosses represent a set of 16×16 simulated pupil centers from a remote eye tracker. In these simulations, the eye-camera location (in millimeters) related to the world coordinate system (i.e., the bottom-center of the screen) were: (**A**) (−250,400,0); and (**B**) (250,0,0).

**Figure 3:**
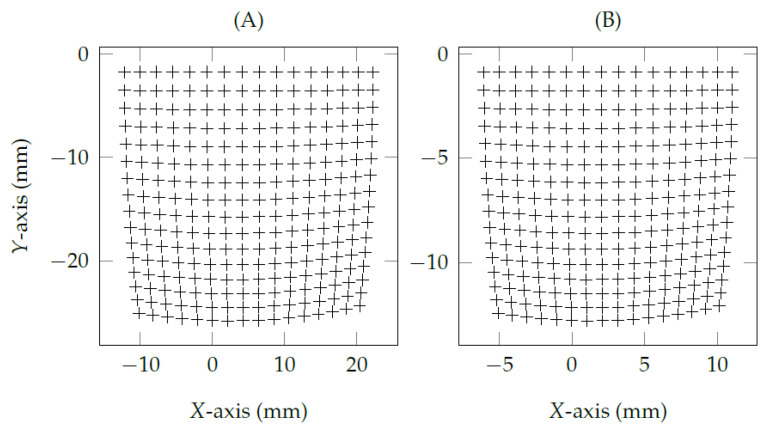
The eye-camera aligned with the eyes’s optical axis and moving in depth. The crosses represent a set of 16×16 simulated pupil centers from a remote eye tracker. In these simulations, the eye-camera location (in millimeters) related to the world coordinate system (i.e., the bottom-center of the screen) were: (**A**) (0,350,0); and (**B**) (0,350,−550).

The shape and coordinates of the eye feature distribution change according to distinct eye-camera locations. Figure 2 shows that the eye-camera displacements around *x*- and *y*-axes have a strong influence on the shape of the eye feature distribution. The larger the β angle, the higher the eccentricity of the eye feature distribution. On the other hand, Figure 3 shows the same shape for two distinct eye feature distributions because these examples use fixed *x*- and *y*- coordinates aligned with $O_{c}$ (i.e., $\beta_{x}=\beta_{y}=0^{\circ }$), while the eye-camera moves in depth from 550 mm to 1100 mm regarding the user’s position. In this condition, the eye-camera displacements in-depth keeps the shape of the eye feature distribution and substantially changes its scale. Figure 3A shows that the scale is twice as big as Figure 3B for both *x*- and *y*-axes.

**Figure 4:**
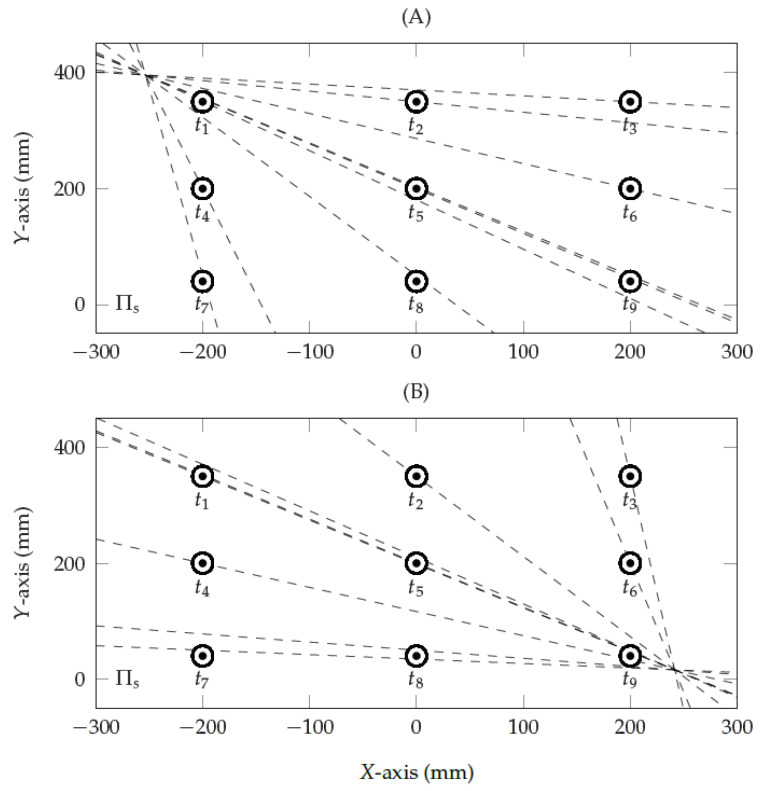
The epipolar geometry describes the eye-camera location in an eye tracker setup. The dots represent a set of 3×3 simulated targets of the gaze-mapping calibration. The epipolar lines pass through each calibration target and intercept at a common point, representing the eye-camera location related to the screen. In these simulations, the 3D eye-camera locations were (**A**) (−250,400,0) and (**B**) (250,0,0).

The proposed eye-camera location compensation method aims to reshape the eye feature distribution to achieve a similar result as virtually aligning the eye-camera as close as possible to the optical axis ($\beta <5^{\circ }$). The method works under the assumption that the eye feature distribution coincides in a plane called the eye plane ($\Pi_{e}$), and all viewed targets and their respective gaze estimations are in a plane called the viewed plane ($\Pi_{s}$). Let us assume the eye plane $\Pi_{e}$ and viewed plane $\Pi_{s}$ as a stereo vision system. The epipolar geometry [24] describes the relationship between a point $p_{e}$ on $\Pi_{e}$ and its corresponding point $p_{s}$ on $\Pi_{s}$ that must lie on the epipolar line $l={\left[\begin{array}{ccc} a & b & c \end{array}\right]}^{T}$. The geometric transformation from the eye feature $p_{e}$ to an epipolar line *l* is given by $l=F\cdot p_{e}$, where *F* is the fundamental matrix under the assumption that encapsulates the intrinsic parameters of the eye-camera, and *l* that defines a straight line in 2D based on the general equation of a line ax+by+c=0 [24]. Figure 4 shows the epipolar geometry of a monocular remote eye tracker. The epipolar lines intercept at a common point called epipole *e*, representing the eye-camera location related to $\Pi_{s}$, and F·e=0 gives it. The epipoles in Figure 4A,B coincide with the eye-camera locations used to generate the eye feature distributions shown in Figure 2A,B, respectively.

The proposed eye-camera location compensation method considers the use of a normalized space $\Pi_{n}$ between $\Pi_{e}$ and $\Pi_{s}$ in order to reduce the influence of eye-camera location [4]. The proposed method normalizes the eye feature distribution into a unit square in a range of [−1,+1] using a polynomial regression as defined in Equation (1):

(1) $$\begin{array}{c} x_{n}=a_{0}x_{e}^{2}+a_{1}y_{e}^{2}+a_{2}x_{e}y_{e}+a_{3}x_{e}+a_{4}y_{e}+a_{5}\\ y_{n}=b_{0}x_{e}^{2}+b_{1}y_{e}^{2}+b_{2}x_{e}y_{e}+b_{3}x_{e}+b_{4}y_{e}+b_{5} \end{array},$$

where $a_{i}$ and $b_{i}$ are the unknown coefficients of the second-order polynomial in the *x*- and *y*-axes [6,14,25]. The polynomial requires a minimum of nine corresponding points ($p_{e}^{i}\Leftrightarrow p_{n}^{i},1\leq i\leq 9$) to solve the unknown coefficients $a_{i}$ and $b_{i}$. It is feasible to reuse the same eye feature distribution used in the gaze-mapping calibration, to derive the mapping from $\Pi_{e}$ to $\Pi_{n}$.

**Figure 5:**
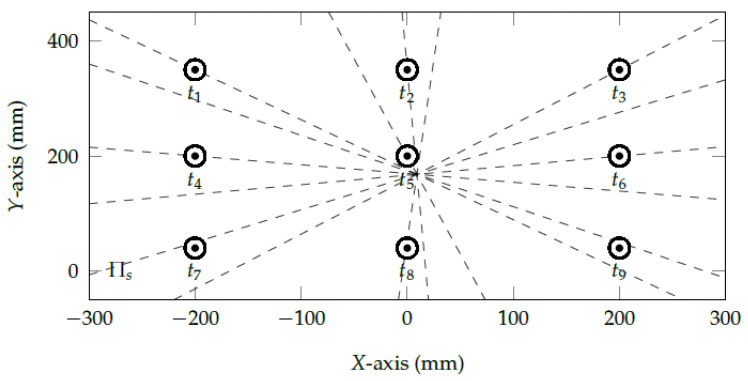
The epipolar geometry between the normalized space $\Pi_{n}$ and the viewed space $\Pi_{s}$. After normalizing the eye-tracking data using a second-order polynomial, the epipole represents the eye-camera location in relation to $\Pi_{s}$ which is very close to the actual center of the viewed plane.

We have evaluated different polynomial regressions [6,7] and geometric transformations [4,5,26] to reshape the eye feature distribution into a normalized space $\Pi_{n}$. Using traditional normalization approaches based on feature scaling would only re-scale the eye feature distribution into a pre-defined range (e.g., min-max normalization). On the other hand, the second-order polynomial regression, shown in Equation (1), changes the scale and reshapes the eye feature distribution into the entire normalized space. Initial tests have shown that using higher-order polynomials [6,7,25] overfit the model and take the epipole at infinity, i.e., epipolar lines become parallel. To better illustrate the proposed method’s effects, Figure 5 shows the epipolar geometry between the normalized eye feature $p_{n}^{i}$ used in the gaze-mapping calibration and the calibration targets $t_{i}$. The normalization based on a second-order polynomial brings the epipole (i.e., virtual camera center) near the center of the screen ($\alpha <5^{\circ }$).

In general, the traditional interpolation-based methods use the geometric transformation $T_{e}^{s}$ to map the eye feature $p_{e}$ into gaze coordinates $p_{s}$ directly. This work proposes using the normalized eye-tracking data $p_{n}$ to estimate the user’s gaze given by $T_{e}^{s}=T_{n}^{s}\circ T_{e}^{n}$. The transformation $T_{n}^{s}$ represents the gaze estimation based on any interpolation-based method, such as the polynomials ($P_{n}^{s}$) [14,27], affine transformations ($A_{n}^{s}$) [5], homographies ($H_{n}^{s}$) [4,28], or cross-ratios ($Cr_{n}^{s}$) [26,29].

Concerning the remote eye tracker setups, it is crucial to use an additional mapping $T_{e}^{g}$ between $\Pi_{e}$ and $\Pi_{n}$ to create a glint normalization space $\Pi_{g}$ which handles the effects of head movements. A primary strategy is to use the reference points from the 3D space (e.g., homography normalization, pupil center-corneal reflection (PCCR)) in order to reduce the head movements’ influence—similarly as previously seen for single [30], dual [14], triple [5], and quad glint normalization approaches [4]. In this case, the proposed method in this work estimates the user’s gaze given by $T_{e}^{s}=T_{n}^{s}\circ T_{g}^{n}\circ T_{e}^{g}$.

### 2.2. Eye Feature Distribution Undistortion Method

This subsection proposes a method to compensate for the distortion in the normalized eye feature distribution. Due to the non-coplanarity between the eye plane $\Pi_{e}$ and the eyeball rotations, the normalized eye feature distribution presents a distortion similar to the barrel effect from camera lenses. The proposed method combines the radial, tangential, and prism distortion equations to model the non-coplanarity error. Therefore, the proposed method undistorts the normalized eye feature distribution and uses the undistorted distribution as the input data for the eye-tracking pipeline.

**Figure 6:**
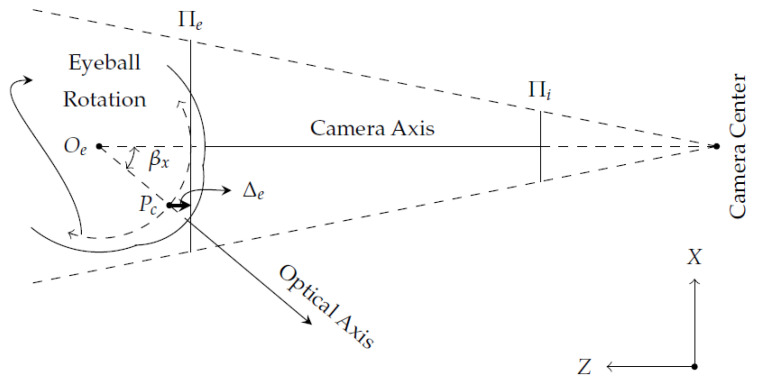
This geometric relationship shows the horizontal eyeball rotation in relation to the eye plane $\Pi_{e}$. The image plane $\Pi_{i}$ represents the captured eye image. The eyeball rotates around a fixed point $O_{e}$, and the maximal angle of rotation is 35 degrees in both right and left directions. The larger the angle β, the higher the error $\Delta_{e}$ between the pupil center $P_{c}$ and the eye plane $\Pi_{e}$.

The eyeball rotates around its center $O_{e}$, moving 35 degrees in both left and right directions within the *x*-axis, and 25 degrees in ascending angle and 30 degrees in descending angle within the *y*-axis [31,32]. In general, interpolation-based eye-tracking methods assume a simplified eye model in which the pupil center $P_{c}$ always coincides with the eye plane $\Pi_{e}$, even over large eye rotations. Assuming a fixed distance between $O_{e}$ and $P_{c}$, when the eyeball rotates, the pupil center will go through a nonlinear path regarding $\Pi_{e}$, as illustrated as a dashed curve in Figure 6.

Given an eye-camera aligned with the eyeball center in both *x*- and *y*-axes ($\beta =0^{\circ }$), the eye-camera captures the eye’s projection onto the image plane $\Pi_{i}$ centered at the camera’s principal point. When the eye’s optical axis points towards the camera center, the pupil center $P_{c}$ coincides with the eye plane $\Pi_{e}$, and the error $\Delta_{e}$ and the angle β are zeros. On the other hand, the error $\Delta_{e}$ increases when the angle β increases due to the non-coplanarity between $\Pi_{e}$ and the eyeball rotations. Therefore, the pupil center $P_{c}$ ray gets displaced radially from its ideal location before hitting the image plane $\Pi_{i}$.

The proposed eye feature distribution undistortion method aims to reduce the influence of the non-coplanarity eye plane in interpolation-based methods. After compensating the eye-camera location, the eye feature distribution presents a systematic pattern on the normalized space. Regardless of the actual eye-camera location in the eye tracker setup, the eye-camera location compensation method reshapes the eye feature distribution in a geometric pattern easily able to learn and understand. The normalized eye feature distribution consists of elliptic iso-contours centered around the camera axis, in which the pupil center coordinates form a structure similar to an ellipsoidal vector.

**Figure 7:**
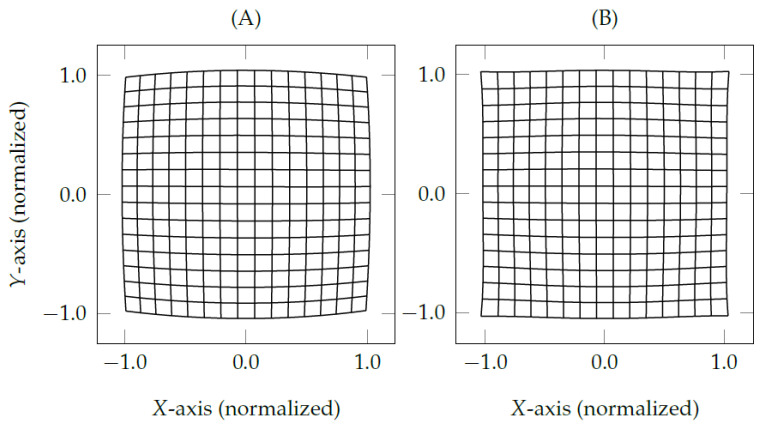
The eye feature distribution on the normalized space $\Pi_{n}$ presents a positive radial distortion (i.e., barrel distortion) available in most camera lenses. The grids represent a set of 16×16 simulated pupil centers from a remote eye tracker with the eye-camera placed at (0,350,0). (**A**) shows the pupil center distribution over the influence of barrel effect, and (**B**) presents the result of the proposed eye feature distribution undistortion method.

Figure 7A shows that the normalized eye feature distribution presents a distortion mostly similar to the one in camera lenses, i.e., fish-eye effect or barrel effect. The grid corners illustrate the relationship between nearest neighbors of 16×16 pupil centers, in which the pupil centers bend more near the edges than the ones near the center of eye feature distribution. The grid in Figure 7A has mostly radial distortion, slightly tangential distortion, and thin prism distortion. The proposed eye feature distribution undistortion method mathematically models the distortion effects the same as the lens properties of calibrated cameras used in OpenCV (available on https://docs.opencv.org, accessed on 21 August 2021). The following equations [33] models the error magnitude $\Delta_{e}={\left(x^{\prime },y^{\prime }\right)}^{T}$ as a function of the normalized eye feature $p_{n}={\left(x_{n},y_{n}\right)}^{T}$.

Equation (2) models the radial distortion ρ in both *x*- and *y*-axes:

(2) $$\rho =\frac{1+k_{1}r^{2}+k_{2}r^{4}+k_{3}r^{6}}{1+k_{4}r^{2}+k_{5}r^{4}+k_{6}r^{6}},$$

where $k_{i}$ are the radial distortion coefficients (1≤i≤6) and $r^{j}={\left(x_{n}^{j}+y_{n}^{j}\right)}^{\frac{1}{2}}$ in which *j* assumes 2, 4, or 6. We assume the radial distortion because straight lines in the eye feature distribution appear to be curved in the normalized plane $\Pi_{n}$. Equation (3) models the tangential distortion τ distinctly in *x*- and *y*-axes to make the eye feature distribution approximately parallel on the normalized plane $\Pi_{n}$:

(3) $$\begin{array}{c} \tau_{x}=2p_{1}x_{n}y_{n}+p_{2}\left(r^{2}+2x_{n}^{2}\right)\\ \tau_{y}=p_{1}\left(r^{2}+2y_{n}^{2}\right)+2p_{2}x_{n}y_{n} \end{array},$$

where $p_{1}$ and $p_{2}$ are tangential distortion coefficients. We assume the tangential distortion because the eye feature distribution seems to be slightly stretched in the normalized plane $\Pi_{n}$. Finally, Equation (4) models the prism distortion ϕ to tilt the eye feature distribution with respect to the normalized plane $\Pi_{n}$:

(4) $$\begin{array}{c} \phi_{x}=s_{1}r^{2}+s_{2}r^{4}\\ \phi_{y}=s_{3}r^{2}+s_{4}r^{4} \end{array},$$

where $s_{k}$ are the prism distortion coefficients (1≤k≤4). We assume the prism distortion because it handles thin imperfections of the eye feature distribution in the normalized plane $\Pi_{n}$. Thus, the sum of radial distortion ρ, tangential distortion τ, and prism distortion ϕ represents the total distortion (i.e., error magnitude $\Delta_{e}$) of the eye feature distribution in the normalized plane $\Pi_{n}$, as expressed in Equation (5):

(5) $$\Delta_{e}=\rho +\tau +\phi .$$

Nonlinear search techniques can quickly solve the distortion coefficients as the error function is well-behaved. Even a small number of point-to-point correspondences give enough information to correct the eye feature distortion. An iterative optimization algorithm (e.g., gradient descent) minimizes the error related to the distance from the normalized eye feature distribution to a squared unit on the normalized space $\Pi_{n}$.

Let $p_{n}={\left(x_{n},y_{n}\right)}^{T}$ be a normalized eye feature in $\Pi_{n}$ without considering the distortion. To compensate the non-coplanarity of the eye plane $\Pi_{e}$, the true normalized eye feature $p_{n}^{*}$ is a function of the estimated normalized eye feature $p_{n}$ and the error magnitude $\Delta_{e}$, as illustrated in Equation (6):

(6) $$p_{n}^{*}=p_{n}\cdot \rho +\tau +\phi .$$

Figure 7B shows the result of the proposed eye feature distribution undistortion method using the same eye-tracking data from the gaze-mapping calibration with nine corresponding points ($p_{n}^{i}\Leftrightarrow p_{s}^{i},1\leq i\leq 9$). In the following, we denote the traditional interpolation-based methods as $T_{e}^{s}$, the methods that use only the proposed eye-camera location compensation as $T_{e}^{s+}$, and the methods that use both proposed eye-camera location compensation and eye feature distribution undistortion as $T_{e}^{s*}$.

### 2.3. Simulated Study

The simulated study aims to statistically evaluate the eye-camera location’s influence on gaze estimation accuracy and identify the most helpful eye-camera location in a real eye tracker device. We have used the et_simul (the original MATLAB source code is available on http://webmail.inb.uni-luebeck.de/inb-toolsdemos/FILES/et-simul-1.01.zip, accessed on 24 October 2020), a MATLAB eye tracker framework to collect simulated eye-tracking data (the source code used to generate the simulated data is available on https://github.com/fabricionarcizo/et_simul/tree/mdpi-vision-2021, accessed on 31 July 2021) from an entirely controlled environment [34]. The simulator allows controlling various settings of a remote or head-mounted eye tracker (e.g., cameras, infrared light sources, viewed plane, targets) and the human ocular system’s parameters (e.g., angle Kappa, aqueous humor’s refractive index, pupil dilation, cornea radius, the distance between eyeball center and pupil center). Therefore, we have used the simulated study to control all noise sources in the eye-tracking pipeline and individually evaluate eye-camera locations’ influence on gaze estimation accuracy.

This study has collected simulated eye-tracking data from 9261 different settings, in which each simulation has used the eye-camera in a distinct and fixed position in the environment. The camera moved in 21×21×21 positions in the three-dimensional space, between −200 mm and 200 mm on the *x*-axis, 50 mm and 350 mm on the *y*-axis, and 0 mm and 400 mm on the *z*-axis. The world coordinate system was at the middle bottom of the viewed plane WCS=(0,0,0), and the simulated monocular eyeball center was aligned to the center of the viewed plane at a distance of 550 mm $O_{e}=\left(0,200,550\right)$.

All simulated data generated in this study are based on a realistic eye model with the standard framework parameters, i.e., a constant refraction index (1.336) and angle Kappa $\left(K_{\alpha }=6^{\circ },K_{\beta }=2^{\circ }\right)$. The viewed plane represents a computer screen of 400×300 mm, and it shows the viewed targets in a range from −200 mm to 200 mm on the *x*-axis, and from 50 mm to 350 mm on the *y*-axis. During each simulation, the eyeball location is kept still while gazing at a uniformly distributed set of 21×21 targets on the viewed plane. The gaze-mapping calibration has used a subset of the viewed targets as a set of nine calibration targets arranged in a 3×3 grid. In total, the simulated study has generated 4,084,101 gaze estimations for each experiment.

### 2.4. User Study

The user study aims to assess the behavior of the proposed methods in real eye-tracking scenarios. This assessment consists of looking at a set of targets linearly distributed on the computer screen and evaluating if it is possible to reduce the gaze error offset regarding traditional interpolation-based eye-tracking methods. The collected real eye-tracking data also created an open-source dataset for eye-tracking studies, which contains the following data: frame number, target ID, timestamp, viewed target coordinates, pupil center, the major/minor axes and angle orientation of fitted ellipse, and four enumerated corneal reflections’ coordinates. We have extracted the eye features from recorded eye videos using a feature-based eye-tracking method (i.e., binarization+fitting ellipse), and the raw data are available on individual annotated text files (CSV).

#### 2.4.1. Design

The evaluation using real eye-tracking data assesses the gaze estimation error from six different scenarios, considering the traditional polynomial ($P_{e}^{s}$) and homography ($H_{e}^{s}$) eye-tracking methods, and the proposed methods to compensate for the eye-camera location ($P_{e}^{s+}$ and $H_{e}^{s+}$) and the eye feature distortion ($P_{e}^{s*}$ and $H_{e}^{s*}$).

#### 2.4.2. Eye-Tracking Data

We have built a remote eye tracker with off-the-shelf components to collect the real eye-tracking data. The collected data contain binocular eye information from 83 participants (166 trials). The dataset contains outliers due to blinks, light reflections, missing glints, and low contrast between the iris and pupil. The valid eye-tracking data used in this study have a mean gaze offset less than or equal to 5 degrees and belong to the 99.7th percentile of all standard deviation. In total, the data analysis presents the assessment using real eye-tracking data from 65 left eyes and 68 right eyes.

#### 2.4.3. Apparatus

The prototype has used one Point Grey Grasshopper3 (GS3-U3-41C6NIR-C) integrated with an infrared global shutter sensor (CMOSIS CMV4000-3E12 NIR), which allows us to collect high-definition images (1600×1200, 4.1 MP) in a frame rate of 150 FPS. The distance between the eye-camera and the user’s eyes was about 20 cm. The eye-camera has used a Navitar Machine Vision c-mount lens (NMV-35M1) of 35 mm (effective focal length) and f/1.4 (aperture). The lens had manual focus, an iris with locking screws, and a field angle of $20.9^{\circ }\times 15.8^{\circ }$. We attached an infrared narrow pass filter (BP850 830–850 nm) between the lens and the camera sensor to improve the contrast of infrared eye images and block any noise from the visible spectrum (e.g., screen reflections). The eye tracker had a 24-inch AOC E2460PHU monitor (240LM00010) with 1920×1080 resolution, widescreen area of 531.36×298.89 mm, and pixel size of 0.27675 mm. We attached a set of 870 nm high-speed infrared emitting diodes (TSFF5510) around each monitor corner. These LEDs helped increase the contrast between the pupil and the iris and create the corneal reflections used to compensate for the head movements.

#### 2.4.4. Participants

We have recruited a sample of 83 volunteer participants (55 males and 28 females) for this experiment. Fifty-five had normal vision, twenty-three wore glasses, and five wore contact lenses. Among the female participants, fifteen wore makeup on their faces or mascara in the eyelashes. The participants were free to blink during data collection, take a rest between the trials, or withdraw from testing at any stage. The participants have used a chin rest to reduce the head movements during the data collection.

#### 2.4.5. Tasks

For each trial, the participant looked at targets arranged in a 5×7 grid in randomized order. The participant has sat approximately 450 mm and orthogonal to the screen. Stimuli showed the target at the same positions and order for 2 s. We have discarded the first and the last 500 milliseconds to remove saccades’ movements between two targets, totaling 5250 collected samples per participant/trial. Among the collected data, the gaze-mapping calibration has used nine targets arranged in a 3×3 grid (8 targets arranged around the screen boundaries and 1 target at the screen center) to calibrate the gaze estimation methods.

#### 2.4.6. Experiment Protocol

First, we have explained the experiment to the participant and obtained her/his signature on the consent document. Afterward, we have made the fine adjustments in the eye tracker components (i.e., infrared light sources, screen, eye-camera, and chin rest) before running the experiment trial. Each participant has experimented twice, the first trial to collect from the right eye and the second one for the left eye. In the end, we have checked the recorded eye-tracking data and interviewed the participant about fatigue or any physical discomfort during the experiment (no participant has made claims about that). On average, the experiment, including two trials, has lasted 7 min and 58 s.

#### 2.4.7. Independent and Dependent Variables

The independent variables are the pupil centers, four glints, and viewed targets. Although the participants have used a chin rest during the data collection, we have normalized the pupil centers using the quad glint normalization approach [4,5] to reduce the head movements’ influence observed in the high-resolution eye images. The two-dimensional target coordinates represent the ground-truth data used to calculate the offset between the estimated gaze and the actual viewed target. The dependent variables include the normalized eye feature and the gaze error offset in pixels and degrees.

#### 2.4.8. Measures

For each viewed target, the remote eye-tracker has collected a sample of 150 eye features, i.e., a total of 871,500 eye features. We have used Kernel Density Estimation (KDE) to calculate the most representative two-dimensional coordinate in each sample. This user study presents the assessment of 5810 eye features based on the binocular information of 83 participants, viewing 35 targets on the screen $\left(2\times 83\times 35\right)$. Initially, the gaze error offset represents the Euclidean distance between the gaze estimation and the viewed target in pixels. The eye-tracking studies usually present the gaze error offset in degrees in the user’s field of view. Therefore, we have calculated the gaze error offset in degrees based on the right-angled triangle, given the screen’s physical pixel size (0.27675 mm) and the distance between the user and the screen (450 mm) [35,36].

#### 2.4.9. Hypotheses

We hypothesize that eye-camera location considerably influences the average accuracy of interpolation-based eye-tracking methods in uncalibrated setups ($H_{1}$). If the eye plane, screen plane, and camera plane are axis-aligned planes, the distance between the eye-camera and the user’s eye will not influence the gaze estimation accuracy because it would not change the eye feature distribution shape ($H_{2}$). Reshaping the eye feature distribution in a normalized plane between the eye plane and the screen plane could obtain similar results as aligning the eye-camera in the eye’s optical axis ($H_{3}$). Therefore, it would be possible to model the non-coplanarity error of the eye plane and the eyeball rotations and corrects the simple planarity assumption in uncalibrated setups ($H_{4}$).

## 3. Results

This section describes a simulated experiment using 9261 different eye tracker settings and a user study with 83 participants to assess the proposed eye-camera location compensation method (see Section 2.1) and the proposed eye feature distribution undistortion method (see Section 2.2). The data analysis evaluates two traditional interpolation-based eye-tracking methods (i.e., polynomial and homography) and their variations using the proposed methods. The evaluation considers the gaze error offset in degrees between the actual viewed targets’ coordinates and the gaze estimations. This assessment aims to evaluate the eye-camera location’s influence (see Section 3.1) and the non-coplanarity of the eye plane (see Section 3.2 and Section 3.3) on the accuracy and precision of interpolation-based gaze estimation methods.

### 3.1. Evaluation of Eye-Camera Location

**Figure 8:**
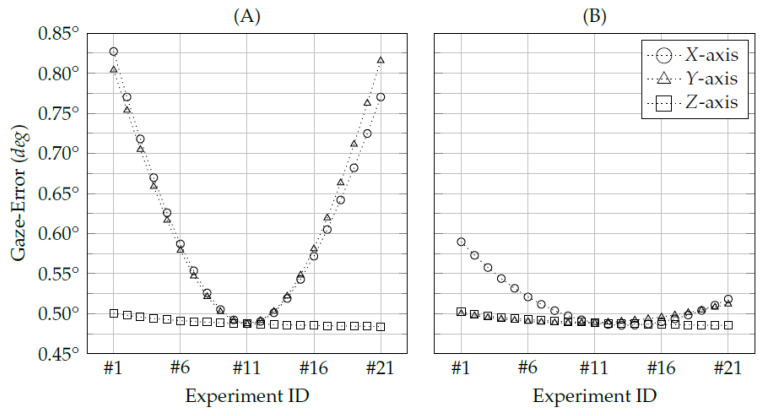
Accuracy as a function of the eye-camera location. The eye-camera has moved to 21 different locations (fixed steps) between the pre-defined ranges, i.e., *x*-axis (from −200 mm to 200 mm), on *y*-axis (from 50 mm to 350 mm), and *z*-axis (0 mm to 400 mm). (**A**) the accuracy of the traditional homography gaze estimation method, and (**B**) the accuracy of the traditional second-order polynomial gaze estimation method.

The first evaluation aims to assess the eye-camera location’s influence on the polynomial-based and homography-based gaze estimation methods. It has used simulated eye-tracking data to evaluate the camera translations individually on *x*-, *y*-, and *z*-axes. This evaluation considers the eye-camera moving to a new position for each experiment while keeping all eye, screen, targets, and eye tracker parameters. It has collected 441 eye-tracking data for each experiment, which has used nine of them to calibrate the gaze estimation method. Figure 8 shows the average accuracies of each experiment while moving the camera on the *x*-axis (from −200 mm to 200 mm, steps of 20 mm), on the *y*-axis (from 50 mm to 350 mm, steps of 15 mm), and *z*-axis (0 mm to 400 mm, steps of 20 mm).

Experiments #11 have achieved the smallest gaze-errors in all trials because the eye-camera was aligned with the screen center and the eyeball center on both *x*- and *y*-axes (α=β=0). The homography-based gaze estimation method has shown gaze-error magnitudes which are larger than the polynomial-based method due to the eye-camera locations, especially the *x*- and *y*-axes movements. Both *x*- and *y*- eye-camera movements have shown systematic errors, similar to a quadratic time function $O(n^{2})$. Using the homography-based method, the *x*-axis variance of gaze-error was $1.07^{\circ }\times 10^{-02}$, and the *y*-axis variance was $1.14^{\circ }\times 10^{-02}$. On the other hand, in the polynomial-based method, the *x*-axis variance was $8.69^{\circ }\times 10^{-04}$, and the *y*-axis variance was $4.44^{\circ }\times 10^{-05}$. Both gaze estimation methods have shown similar behavior in *z*-axis experiments. When the eye-camera moves in-depth, it captures the eye feature distribution at the same view-angle. Therefore, the eye-camera captures the eye features in a similar distribution shape but different coordinates scale, as shown in Figure 3. The variances of *z*-axis experiments using the homography-based method was $2.18^{\circ }\times 10^{-05}$, and using the polynomial-based method was $2.28^{\circ }\times 10^{-05}$.

**Figure 9:**
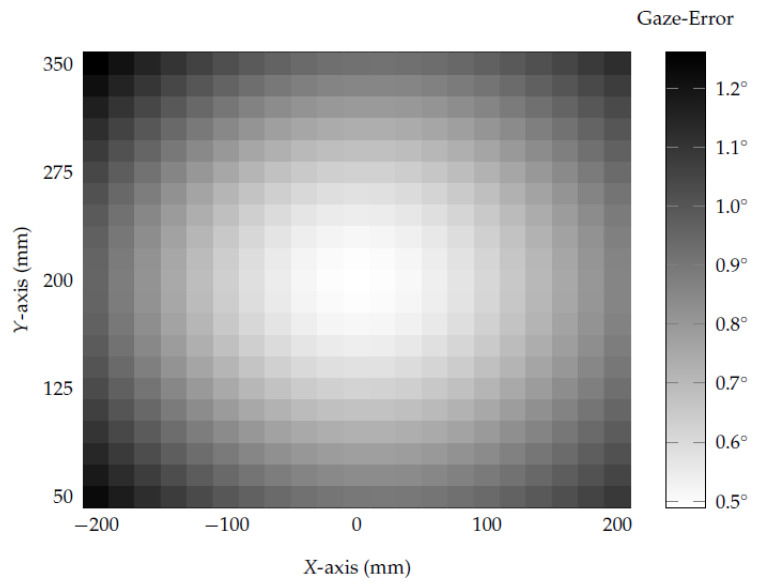
This heatmap illustrates the eye-camera location’s influence on the traditional homography-based gaze estimation method’s accuracy. The eye-camera has moved in a grid of 21×21×21 positions (i.e., 9.261 settings). Each element in this heatmap represents the gaze error average of 21 camera displacements along the *z*-axis. When the optical axis, screen axis, and camera axis are aligned (X=0 mm and Y=200 mm), the gaze error is $0.49^{\circ }$.

Besides evaluating each axis movement individually, this evaluation has combined all eye-camera positions from the first experiments to assess the eye-camera location’s influence in a remote eye tracker setup. This evaluation considers the eye-camera moving on the *x*-axis (from −200 mm to 200 mm), *y*-axis (from 50 mm to 350 mm), and *z*-axis (0 mm to 400 mm), which combines a total of 9261 trials (21×21×21 camera positions in the three-dimensional space). Figure 9 shows the two-dimensional overview of the gaze-error from the traditional homography-based gaze estimation method.

Each grid cell represents the average gaze-error achieved with the eye-camera placed at fixed two-dimensional coordinates (on *x*- and *y*-axes), while the eye-camera moves in depth along the *z*-axis (i.e., the average of 21 gaze estimations). The highest accuracy occurs when the eye-camera is aligned with the eyeball center and screen center, in which the gaze-error is $0.49^{\circ }$. When the angles between the eye-camera and screen axes (i.e., α) and between the eye-camera and optical axes (i.e., β) increase, the gaze-error also increases in quadratic-order, as shown in Figure 8A. The lowest accuracy occurs in the top-left area (X=−200 mm and Y=350 mm), in which the gaze-error is $1.26^{\circ }$. The overall variance of the traditional homography-based gaze estimation method was $8.11^{\circ }\times 10^{-02}$, and the traditional polynomial-based method was $5.92^{\circ }\times 10^{-03}$.

### 3.2. Evaluation of Proposed Methods Using Simulated Data

**Figure 10:**
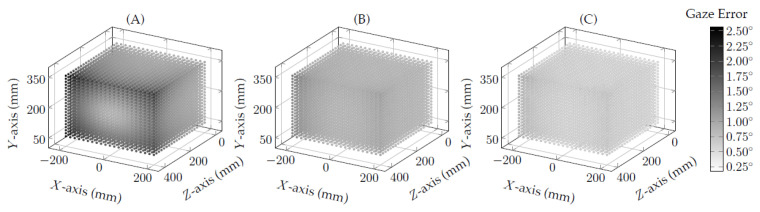
A three-dimensional overview of the eye-camera location’s influence on the homography-based gaze estimation methods. Each dot represents an eye-camera location in the three-dimensional space, and each scatter plot represents a set of 9261 eye-camera locations. (**A**) shows the gaze errors achieved by the traditional homography-based method, which presents the highest gaze error ($2.56^{\circ }$) in the simulated study at location X=−200 mm, Y=350 mm, and Z=400 mm, (**B**) illustrates the improvements achieved with the eye-camera location compensation method, and (**C**) presents the results of the eye feature distribution undistortion method, which achieves the best gaze estimation accuracy ($0.18^{\circ }$) at location X=0 mm, Y=200 mm and Z=[0 mm ,400 mm].

The evaluation initially assessed the proposed eye-camera compensation method and the proposed eye feature undistortion method using simulated eye-tracking data. This evaluation aimed to test and prove our hypotheses $H_{3}$ and $H_{4}$ in a scenario that avoids the influence of several sources of noise (e.g., light conditions, misclassification in the eye feature detection, blinks, among others). In the following, the data analysis has used the same eye-tracking data collected during the previous evaluation (see Section 3.1) to measure the improvements in the gaze estimation accuracy when using both the proposed compensation methods compared to the traditional interpolation-based gaze estimation methods. Figure 10 shows the three-dimensional overview of the gaze-error from the homography-based gaze estimation methods (i.e., $H_{e}^{s}$, $H_{e}^{s+}$, and $H_{e}^{s*}$).

The scatter plot represents each eye-camera location in the world coordinate (i.e., the bottom-center of the screen). The lighter dot colors represent high-accuracy gaze estimations, and the darker dot colors represent large gaze errors. Figure 10A shows the average gaze-error of the traditional homography-based gaze estimation method, i.e., it is a three-dimensional overview of gaze errors represented in Figure 9. In this experiment, the gaze-error distribution is in the range from $0.48^{\circ }$ to $2.56^{\circ }$. Figure 10B shows the improvements achieved using the proposed eye-camera location compensation method. The scatter plot is lighter than the one represented in Figure 10A, and it shows the gaze-error distribution is in the range from $0.48^{\circ }$ to $1.29^{\circ }$. Finally, Figure 10C shows the results of the proposed eye feature distribution undistortion method. This method has achieved the best gaze estimation accuracy in this evaluation. Its gaze-error distribution is in the range from $0.18^{\circ }$ to $0.75^{\circ }$.

**Figure 11:**
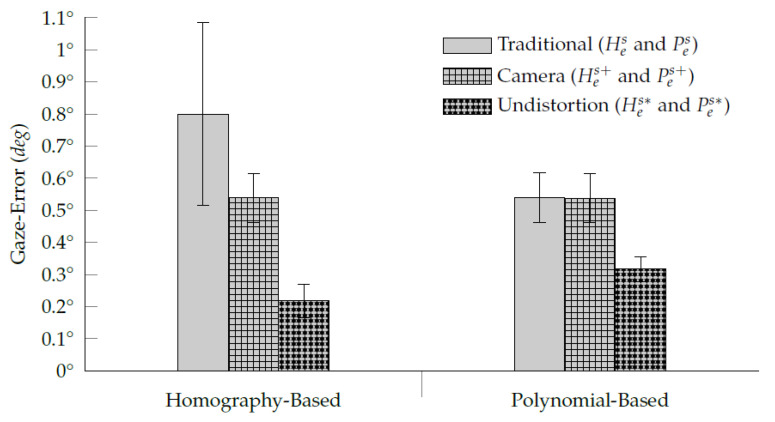
The average gaze-error distribution of simulated eye-tracking data analysis. The bar plots show the improvements achieved with the proposed eye-camera location compensation ($H_{e}^{s+}$ and $P_{e}^{s+}$), and proposed eye feature distribution undistortion ($H_{e}^{s*}$ and $P_{e}^{s*}$) over the traditional interpolation-based gaze estimation methods ($H_{e}^{s}$ and $P_{e}^{s}$). The large error bar in the traditional homography-based method $H_{e}^{s}$ is due to is sensitivity to the eye-camera location’s influence.

Figure 11 shows the average gaze-error distribution of each assessed interpolation-based gaze estimation method. As expected, the traditional homography-based method is the one that presents the highest variance due to its sensitivity to the eye-camera location’s influence. For this reason, the eye-camera compensation method was more efficient in the homography-based method than in the polynomial-based one. Although a slight difference (<0.01${}^{\circ }$) between the traditional polynomial-based method and its eye-camera location compensation results, the eye feature undistortion method requires eye-camera compensation before correcting the eye feature distribution distortion. The eye feature undistortion method using homography-based and polynomial-based eye-tracking data has achieved the best gaze estimation accuracy. Their gaze-error distributions present a mean of $\bar{H_{e}^{s*}}=0.22^{\circ }\pm 0.05^{\circ }$ and $\bar{P_{e}^{s*}}=0.37^{\circ }\pm 0.04^{\circ }$, respectively.

**Figure 12:**
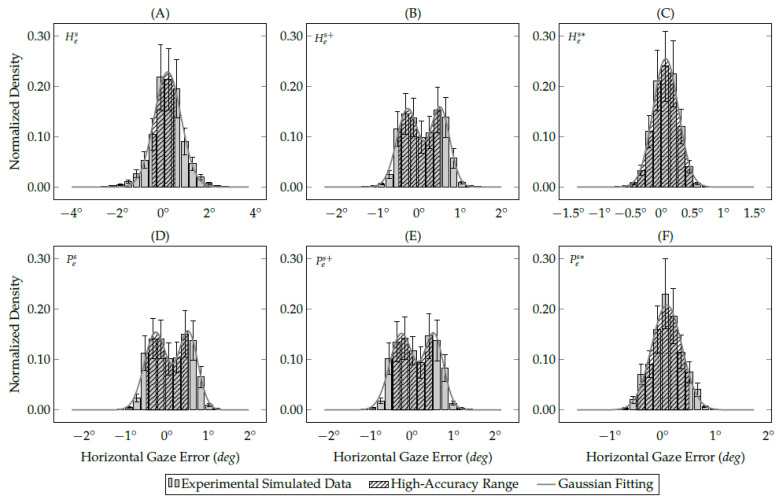
The histograms represent the gaze-error offset on the *x*-axis of all eye-tracking data collected during the simulated study. The areas delimited with northeast lines represent the high-accuracy gaze estimations, in which the (**A**) traditional homography gaze estimation method achieved 58%; (**B**) the homography gaze estimation method with the eye-camera location compensation achieved 64%; (**C**) the homography gaze estimation method with both camera location and distortion compensations achieved 98%; (**D**) traditional polynomial gaze estimation method achieved 64%; (**E**) polynomial gaze estimation method with the eye-camera location compensation achieved 63%; (**F**) polynomial gaze estimation method with both camera location and distortion compensations achieved 91%.

**Figure 13:**
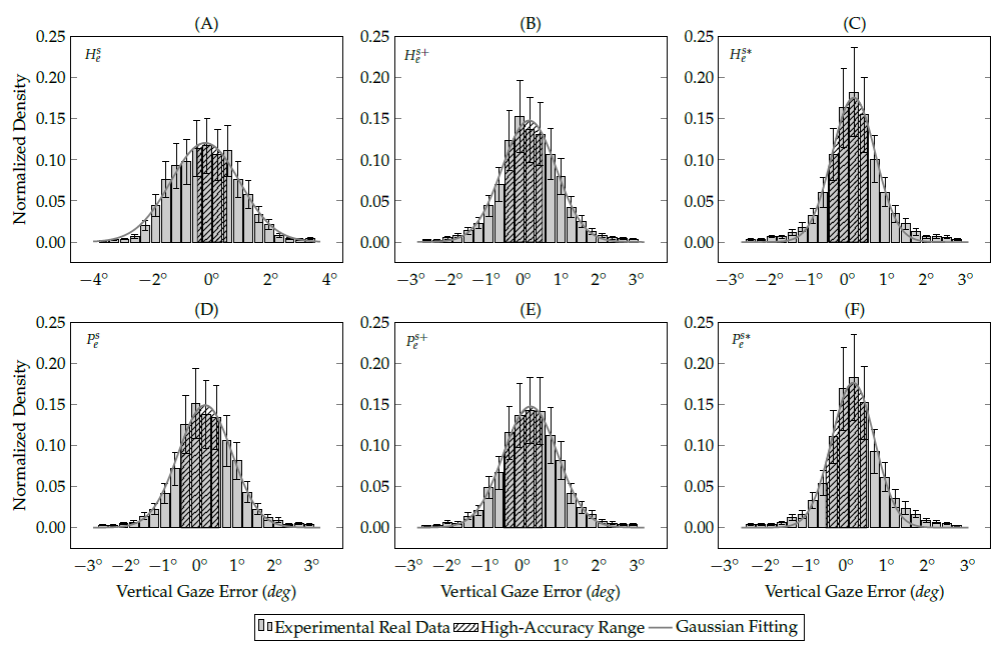
The histograms represent the gaze-error offset on the *y*-axis (without outliers) of the eye-tracking data collected during the user study. The areas delimited with northeast lines represent the high-accuracy gaze estimation, in which (**A**) traditional homography gaze estimation method achieved 32%; (**B**) homography gaze estimation method with the eye-camera location compensation achieved 50%; (**C**) homography gaze estimation method with both camera location and distortion compensations achieved 62%; (**D**) traditional polynomial gaze estimation method achieved 50%; (**E**) polynomial gaze estimation method with the eye-camera location compensation achieved 50%; (**F**) polynomial gaze estimation method with both camera location and distortion compensations achieved 63%.

The following data analysis computes a Gaussian fit over the discrete eye-tracking data to calculate the probability of getting a single gaze estimation between $-0.5^{\circ }$ and $0.5^{\circ }$ (the high-accuracy range) through the Gaussian probability density function (PDF). Both simulated and real eye-tracking data follow a normal distribution, as shown in Figure 12 and Figure 13. Therefore, the data analysis evaluates each experimental data using the Gaussian probability density function, as illustrated in Equation (7):

(7) $$p_{G}\left(x;\mu ,\sigma \right)=\frac{1}{\sigma \sqrt{2\pi }}\exp\left[-\frac{1}{2}{\left(\frac{x-\mu }{\sigma }\right)}^{2}\right].$$

This equation is a continuous function that describes the probability of obtaining a gaze estimation in a random observation from an eye feature distribution with parameters mean (μ) and standard deviation (σ). Figure 12 shows the gaze-error distribution on the *x*-axis of simulated eye-tracking data. In the normalized histogram, the bin height represents the proportion (probability) of gaze estimations that are between the bin’s lower and upper limits. Therefore, the sum of all bins in the histogram and the area under the Gaussian curve are equal to 1. Equation (8) models the Gaussian integral (a.k.a., Euler–Poisson integral) over the entire Gaussian fitting line:

(8) $$I_{G}=\int_{-\infty }^{\infty }p_{G}\left(x;\mu ,\sigma \right)dx=1.$$

Figure 12 also shows the Gaussian functions (the solid gray lines) that fit the discrete gaze-error distributions. The area sizes of Gaussian distributions are equal to 1 in both single- and multi-peak Gaussian functions. The Gaussian integral between $-0.5^{\circ }$ and $0.5^{\circ }$ (defined by the northeast lines) represents the high-accuracy range and helps us to understand the improvements achieved with the methods proposed in this study. The larger the area defined by the northeast lines, the better is the gaze estimation accuracy and precision. Table 1 shows the Gaussian probability density of simulated gaze-error from the evaluated interpolation-based gaze estimation methods. Figure 12 shows only the Gaussian distribution of the *x*-axis because this axis has shown the highest variance among the assessed methods using simulated data. This is on the contrary to the *z*-axis, in which all simulated gaze estimations are between $-0.5^{\circ }$ and $0.5^{\circ }$, as shown in Table 1.

### 3.3. Evaluation of Proposed Methods Using Real Data

The eye-tracking dataset collected during the user study is smaller than the one from the simulated study. In total, the real eye-tracking experiments contain 871,500 gaze estimations, compared to 4,084,101 collected for the simulated one. Nevertheless, the real eye-tracking data also follow the normal distribution. The 83 participants have looked at a set of 35 targets distributed in a 7×5 grid. The user study is based on a sample of 150 eye features for each viewed target in a single second, i.e., 5250 eye features collected for each trial/experiment. The data analysis uses KDE to calculate the most representative two-dimensional coordinate $\left(x_{i},y_{i}\right)$ among the collected sample of 150 eye features. KDE uses the Gaussian PDF (see Equation (7)) to estimate kernel density and optimize bandwidth using the collected eye features. The KDE curve’s highest value is the input eye feature used to estimate the user’s gaze. It means that, for each trial, the data analysis reduced the number of assessed eye-tracking data from 5250 to 35 samples.

The data analysis using real eye-tracking data has binocular eye information from two trials per participant, the first from the right eye and the second from the left eye. Thirty-three trials were discarded due to problems during the data collection and eye feature extraction stages. The outliers from this dataset were also discarded, i.e., gaze-error higher than $5^{\circ }$ and the gaze estimation above three times the standard deviation. The number of assessed eye features from the real eye-tracking dataset changes according to the evaluated interpolation-based gaze estimation method. Therefore, the data analysis has used 5488±32 eye features on average.

Figure 13 shows the gaze-error distribution on the *y*-axis of real eye-tracking data. In the user study, the gaze-error variance on the *y*-axis is more significant than the *x*-axis because of the eye-camera alignment. The eye tracker prototype placed the eye-camera in front of the user’s eyes aligned on the *x*-axis, and with a sizeable down offset on the *y*-axis. Table 2 shows a smaller variance in the gaze-error on the *x*-axis than on the *y*-axis. In the same way as simulated experiments, the number of gaze estimations between $-0.5^{\circ }$ and $0.5^{\circ }$ increases using the methods proposed in this study.

Table 2 shows the Gaussian probability density of real gaze-error from the evaluated interpolation-based methods. In Figure 13, the areas delimited with northeast lines represent the high-accuracy range. The traditional homography-based method presents the smallest area because of its sensitivity to the eye-camera location. In this case, 32% of gaze estimations are between $\pm 0.5^{\circ }$. On the other hand, the eye feature distribution undistortion method was the most useful for both homography-based and polynomial-based methods because it increases the high-accuracy area to 62% and 63%, respectively.

**Figure 14:**
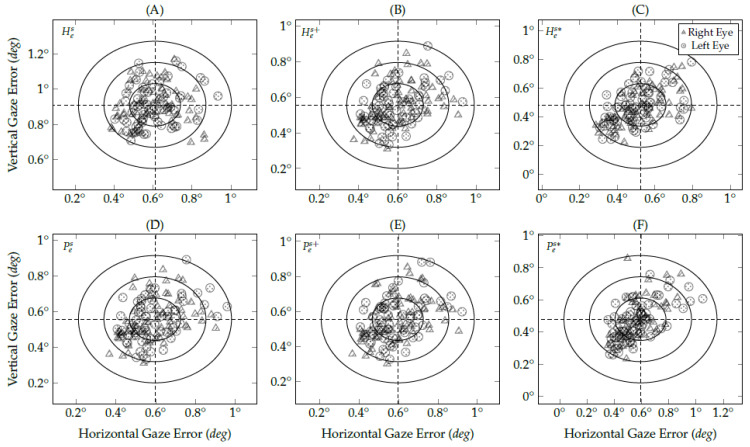
An overview of user study results considering two distinct classes, the gaze estimation from the left and right eye. The three circles in each scatter plot represent the 68–95–99.7 rule of a normal distribution. This figure shows the gaze estimations from (**A**) a traditional homography gaze estimation method; (**B**) a homography gaze estimation method with the eye-camera location compensation; (**C**) a homography gaze estimation method with both camera location and distortion compensations; (**D**) a traditional second-order polynomial gaze estimation method; (**E**) a polynomial gaze estimation method with the eye-camera location compensation; (**F**) a polynomial gaze estimation method with both camera location and distortion compensations.

The data analysis has used the actual gaze-error based on the Euclidean distance between the ground-truth data (i.e., the coordinates of viewed targets) and the measured data (i.e., the gaze estimations). However, it is common in eye-tracking studies to measure eye trackers’ accuracy and precision using the absolute error. Figure 14 shows the reliability offset of gaze estimation distribution using the absolute accuracy from real eye-tracking data (both right and left eyes). One of the primary differences between using the actual and absolute gaze-error is the mean accuracy. The actual mean accuracy tends to $0^{\circ }$ on both *x*- and *y*-axes because the gaze estimation distribution follows the normal distribution, as illustrated in Figure 12 and Figure 13. On the other hand, the mean absolute gaze-error shows the overall magnitude of the center of gaze estimations distribution regarding the actual viewed target.

The plot axes in Figure 14 show the mean absolute gaze-error of evaluated interpolation-based methods. The three circles in the reliability offset distribution plots represent the 68–95–99.7 rule of a normal distribution. Ideally, the dashed lines should be as close as possible to $0^{\circ }$ in each axis. The vertical gaze-error in the traditional homography-based method is $0.91^{\circ }\pm 0.12^{\circ }$, i.e., the highest vertical gaze-error among the evaluated methods. The other five methods present vertical gaze errors lower than $0.56^{\circ }\pm 0.13^{\circ }$. The proposed eye feature distribution undistortion method using homography-based eye-tracking data presents the best accuracy in both axes, which has achieved $G_{X}=0.52^{\circ }\pm 0.14^{\circ }$ and $G_{Y}=0.48^{\circ }\pm 0.15^{\circ }$. The other evaluated methods present similar horizontal gaze-error of about $0.60^{\circ }\pm 0.14^{\circ }$. The proposed eye feature distribution undistortion method using homography-based eye-tracking data brings most of the data to the 68th and 95th percentiles of all standard deviation.

## 4. Discussion

Our results indicate that the accuracy of interpolation-based eye-tracking methods can decay according to the eye-camera location. The primary reason is a deformation in eye feature distribution when the eye-camera moves far from the eye’s optical axis combined with the non-coplanarity of the eye plane $\Pi_{e}$. The objective of our experiments was to evaluate an eye-camera location compensation method that reshapes the eye feature distribution as an approximation of the best eye-camera location without additional information (e.g., intrinsic or extrinsic parameters) from the camera in uncalibrated setups. The targets from the gaze-mapping calibration provide enough information to realign the eye feature in a normalized plane $\Pi_{n}$ and make the pupil center distribution highly smooth. From the experiments, we have obtained different conclusions:

1. Assuming the eye plane $\Pi_{e}$ and the viewed plane $\Pi_{s}$ as a stereo vision system, it is possible to use the epipolar geometry to estimate the eye-camera location in an uncalibrated setup.
2. The second-order polynomial was the one that best compensates for the eye-camera location. We have tested high-order polynomials as well; however, they overfit the model and take the epipole (that represents the virtual eye-camera location) to the infinity, i.e., the epipolar lines become parallel.
3. When the eye-camera is on the eye’s optical axis and moves in depth (*z*-axis), the shape of the eye feature distribution keeps the same while changing its scales on both *x*- and *y*-axes. It means the eye-camera location compensation method must realign the camera only on *x*- and *y*-coordinates in the three-dimensional space.
4. Due to the eye-camera location, the homography-based methods have gaze-error magnitudes more significant than the interpolation-based methods.
5. The proposed methods most benefit uncalibrated setups because it is not required to understand the geometry and the locations of the eye tracker components to reduce the negative influence of large α and β angles of the eye-camera’s optical axis into the gaze estimation.
6. Both proposed methods improve the accuracy of interpolation-based eye-tracking methods using the same eye-tracking data from the gaze-mapping calibration. However, the proposed eye feature distribution undistortion method would benefit from gaining further user data, such as using more calibration data or combining with a recalibration procedure.

The proposed methods are suitable for RET and HMET, uncalibrated, partially and fully calibrated setups, and commercial and non-commercial eye trackers. They complement the traditional interpolation-based methods because, in the eye-tracking pipeline, the proposed methods perform preprocessing geometric transformations to correct the eye feature distribution before the gaze-mapping calibration and gaze estimation. Points in the normalized space $\Pi_{n}$ represent the pure eye feature distribution (e.g., pupil centers) mapped directly from the eye space $\Pi_{e}$. The normalized space $\Pi_{n}$ directly models the optical axis, but it suffers the influence of head movements. However, in a remote setup, it is possible to reduce the influence of natural head movements by combining the proposed methods with a glint normalization approach [4,5,14,30,37,38]. The offset between the optical and visual axes (i.e., the angle Kappa) corresponds to translations in normalized space $\Pi_{n}$ [4]. The angle Kappa is modeled implicitly through $T_{n}^{s}$ by a gaze-mapping calibration. The proposed methods are also helpful for HMET [10,39,40] and head-mounted displays (HMD) [11,41] because they can virtually align the eye-camera in the eye’s optical axis without disturbing the user’s field of view and, at the same time, improve the gaze estimation accuracy.

Another significant contribution of this study is the method to undistort the eye feature distribution and reduce the influence of the non-coplanarity of the eye plane $\Pi_{e}$. It applies the same technique used to correct the lens distortion in computer vision applications. After correcting the camera location, the eye feature distribution on the normalized space $\Pi_{n}$ always presents a barrel distortion (typically $k_{1}<0$) or a pincushion distortion on the contrary (typically $k_{1}>0$) independent of the eye-camera location. The proposed eye feature distribution undistortion method requires placing the calibration targets at the viewed plane’s boundaries to prevent the rectified eye feature from blowing up. In the case of using a more reliable gaze-mapping calibration, e.g., smooth-pursuit-based calibration for RET [42,43] or HMET [21,44,45], the only requirement is to move the calibration target around the entire viewed plane to ensure that the undistortion method learns how to undistort the normalized eye feature distribution correctly.

The data analysis opens up a new threshold to measure high-accuracy in gaze estimation methods, further than the traditional measurement based on mean absolute error up to $0.5^{\circ }$. Our study uses the Gaussian PDF to calculate the probability of a gaze estimation is between $-0.5^{\circ }$ and $0.5^{\circ }$. In a simulated environment, the proposed methods increase the high-accuracy gaze estimation range from 74% to 99% in homography-based methods and from 82% to 96% in interpolation-based methods. In a real scenario, gaze estimation ranges between $-0.5^{\circ }$ and $0.5^{\circ }$ increases from 41% to 57% in homography-based methods and from 49% to 60% in interpolation-based methods. The Gaussian analysis aims to test the probability of the experiment’s success. Therefore, the data analysis has shown a similarity between the simulated and real eye-tracking data since the Gaussian analysis successfully tested the substantial majority of the collected data. It is essential to mention that we have extracted the eye feature from the captured eye images using basic image analysis algorithms (i.e., binarization+fitting ellipse). Using more advanced techniques to extract the eye features [40,46,47,48], the proposed methods could perform better regarding the number of gaze estimations in the high-accuracy range.

## 5. Conclusions

Starting from the traditional interpolation-based gaze estimation methods, we have studied the influence of the eye-camera location in uncalibrated setups and proposed two methods to improve the gaze estimation accuracy. The simulated study evaluates the influence of eye-camera location individually on *x*-, *y*-, and *z*-axes by moving the eye-camera in 21×21×21 different locations in the three-dimensional space. Geometrical analyses of eye-camera location demonstrate that the larger the angles between the eye-camera and the computer screen (α) and the eye-camera and the eye’s optical axis (β), the higher the magnitude of gaze-error. In the traditional homography-based method, the gaze-error increases in quadratic-order in both *x*- and *y*-coordinates. This study also shows that we can achieve high-accuracy gaze estimation with the eye-camera physically aligned to the center of the user’s eyes and the viewed plane. As the physical alignment is not feasible in most eye tracker devices, we proposed to use a normalized space ($\Pi_{n}$) between the eye plane ($\Pi_{e}$) and the viewed plane ($\Pi_{s}$) to obtain similar results as in the physical alignment, even without any information about the intrinsic and extrinsic parameters of the eye-camera. With the eye-camera location compensation method, the eye feature distribution presents a similar shape independent of the eye-camera location. Therefore, we use the simplest lens distortion model to undistort the eye feature distribution and compensate for the simple planarity assumption in uncalibrated setups. The statistical analysis using the Gaussian probability density function reported here found that the proposed methods increase the number of gaze estimations between the range $-0.5^{\circ }$ and $0.5^{\circ }$ in both simulated and user studies.

As future studies, we propose to evaluate the use of a convolutional neural network (CNN) and Deep Learning Models (DLP) to estimate and compensate the locations of the eye-camera, the eyeball center, and the computer screen based only on the eye-tracking data collected during the gaze-mapping calibration. The objective should be to compare the CNN and DLP with the results obtained in this paper and increase the number of gaze estimations in the range of $\pm 0.5^{\circ }$. From now, our main objective is to extend the proposed methods as an alternative to correct the parallax error in uncalibrated head-mounted eye trackers. As the parallax error occurs due to the optical axes of the user’s eye and the eye tracker cameras are not aligned, the results obtained in this paper can be used to correct the parallax error uncalibrated setups. We also aim to evaluate the influence of infrared light sources’ location on gaze estimation accuracy individually. Our last future work is to increase the EyeInfo dataset to add eye-tracking data from head-mounted eye trackers and commercial remote eye trackers.

## Figures and Tables

**Table 1:** The Gaussian PDF of simulated gaze estimations between $-0.5^{\circ }$ and $0.5^{\circ }$.

Methods	Gaze${}_{X}$	Gaze${}_{Y}$	Gaze${}_{Z}$	Average
$H_{e}^{s}$	0.58	0.63	1.00	0.74
$H_{e}^{s+}$	0.64	0.84	1.00	0.83
$H_{e}^{s*}$	0.98	1.00	1.00	0.99
$P_{e}^{s}$	0.64	0.83	1.00	0.82
$P_{e}^{s+}$	0.63	0.84	1.00	0.82
$P_{e}^{s*}$	0.91	0.98	1.00	0.96

**Table 2:** The Gaussian PDF of real gaze estimations between $-0.5^{\circ }$ and $0.5^{\circ }$.

Methods	Gaze${}_{X}$	Gaze${}_{Y}$	Average
$H_{e}^{s}$	0.50	0.32	0.41
$H_{e}^{s+}$	0.50	0.50	0.50
$H_{e}^{s*}$	0.51	0.62	0.57
$P_{e}^{s}$	0.47	0.50	0.49
$P_{e}^{s+}$	0.49	0.50	0.50
$P_{e}^{s*}$	0.55	0.63	0.60

## Data Availability

Code for running the experiment, and all the data and analysis scripts, are accessible in an open access repository: https://github.com/fabricionarcizo/eye-tracking-data (accessed on 7 July 2021).

## Abbreviations

The following abbreviations are used in this manuscript:

PoR	Point-of-Regard
RET	Remote Eye Trackers
HMET	Head-Mounted Eye Trackers
GDPR	Data Protection Regulation
PCCR	Pupil Center-Corneal Reflection
KDE	Kernel Density Estimation
WCS	World Coordinate System
PDF	Gaussian Probability Density Function
HMD	Head-Mounted Displays
CNN	Convolutional Neural Networks
DLM	Deep Learning Models
LoS	Line of Sight
DoF	Degrees of Freedom
OLS	Ordinary Least Squares

## Appendix A. Gaze Estimation Methods

Images from the user’s eyes are valuable data that provide information about the user’s eyes activities, and they are the primary eye-tracking data used for video-based eye-tracking methods. Analyzing eye images makes it possible to identify different human ocular behaviors, such as blinks, fixations, saccades, microsaccades, smooth pursuits, quiet eyes, and gaze. Gaze estimation methods use a mathematical model to calculate an approximation of an individual’s highest point of interest or focus of attention in the field of view. According to the kind of input eye-tracking data, the taxonomy of gaze estimation methods consists of appearance-based and feature-based.

### Appendix A.1. Appearance-Based Gaze Estimation Methods

Appearance-based gaze estimation methods analyze the entire eye image to estimate a coordinate in the viewed plane directly. These methods learn about eye movements behavior, specifically the primary focus of attention in a bidimensional plane. The training procedure of an appearance-based method requires a dataset with a massive sample of eye images that include countless eye appearance variations. In general, appearance-based methods are more robust in estimating the user’s gaze, even using low-resolution eye images. On the other hand, these methods are more sensitive to noise, such as head movements in RET or device slippage in HMET. In recent years, appearance-based gaze estimation methods have become more popular due to increased processing power and improvements in computer vision techniques. Recent research studies have shown good progress, especially in using convolutional neural networks [49,50,51] and deep learning models [52,53] to estimate the user’s gaze with high accuracy.

### Appendix A.2. Feature-Based Gaze Estimation Methods

Feature-based gaze estimation methods extract some external eye features from the eye images (e.g., pupil center, iris center, corneal reflections, eye corners, iris boundary) and use them to estimate the user’s gaze in the viewed plane. These methods require an initial gaze-mapping calibration procedure, i.e., a regression analysis for estimating the relationships between corresponding points from the eye image and the viewed plane. Feature-based methods are less sensitive to environmental light variations and more robust to head movements. For example, the corneal reflections (a.k.a. glints) are good reference points to identify the relationship between the user’s face and the viewed plane in the three-dimensional space (even in uncalibrated setups). Eye features allow us to estimate the Point-of-Regard in a viewed two-dimensional plane using an interpolation-based method or the Line-of-Sight (LoS) (a.k.a. visual axis) in the three-dimensional space using a model-based method. Interpolation-based methods are attractive due to their relative simplicity of implementation, robustness, and accuracy achieved.

Homographic mapping [24,54] is an excellent example for a robust and accurate interpolation-based gaze estimation method [4,28]. Homography defines a planar projective mapping between two distinct planes $\Pi_{A}$ and $\Pi_{B}$. In other words, homography maps a point $p_{A}$ from plane $\Pi_{A}$ to its corresponding point $p_{B}$ in $\Pi_{B}$. Let assume the eye feature distribution at the eye plane $\Pi_{e}$ and their corresponding PoR at the viewed plane $\Pi_{s}$. It is possible to calculate the projective transformation between $\Pi_{e}$ and $\Pi_{s}$ through a homographic mapping $H_{e}^{s}$, i.e., a non-singular 3×3 matrix as defined in Equation (A1):

(A1) $$\begin{array}{cc} H_{e}^{s} & =\left(\begin{array}{ccc} s\cos(\theta ) & s\sin(\theta ) & t_{x}\\ -s\sin(\theta ) & s\cos(\theta ) & t_{y}\\ 0 & 0 & 1 \end{array}\right)\left(\begin{array}{ccc} \frac{1}{b} & 0 & 0\\ 0 & 1 & 0\\ 0 & 0 & 1 \end{array}\right)\left(\begin{array}{ccc} 1 & -\tan(\alpha ) & 0\\ 0 & 1 & 0\\ 0 & 0 & 1 \end{array}\right)\left(\begin{array}{ccc} 1 & 0 & 0\\ 0 & 1 & 0\\ l_{x} & l_{y} & 1 \end{array}\right)\\ \; & =\left(\begin{array}{ccc} h_{11} & h_{12} & h_{13}\\ h_{21} & h_{22} & h_{23}\\ h_{31} & h_{32} & h_{33} \end{array}\right) \end{array},$$

where the first matrix is a two-dimensional rigid transformation, the second is an anisotropic scaling transformation, the third is a skew transformation, and the last is a projective transformation. There are eight independent ratios amongst the nine variables of $H_{e}^{s}$ [24], i.e., homography is a planar projective transformation with 8 Degrees of Freedom (DoF), namely: 1 rotation (θ), 2 translations ($t_{x}$ and $t_{y}$), 1 isotropic scaling (*s*), 1 anisotropic scaling (*b*), 1 skew (α), and 2 perspective shortening ($l_{x}$ and $l_{y}$) [55]. In the gaze-mapping calibration, each pair of corresponding points generates two constraints, and thus a minimum of four corresponding points are enough to solve for $H_{e}^{s}$. After the calibration procedure, it is possible to estimate the user’s gaze through a simple matrix multiplication as $p_{s}=H_{e}^{s}\times p_{e}$, in which $p_{e}={\left[\begin{array}{ccc} x_{e} & y_{e} & 1 \end{array}\right]}^{T}$ is the eye feature in homogenous coordinates and $p_{s}$ is the gaze estimation in the viewed plane.

A well-known application for interpolation-based gaze estimation methods is using general-purpose polynomials regression with unknown coefficients. The gaze-mapping calibration collects the eye-tracking data used to adjust the polynomial coefficients through some numerical fitting process, such as linear regression or Ordinary Least Squares (OLS), which minimizes the sum of squared residuals between the eye feature and viewed target coordinates. Eye-tracking methods widely use second-order polynomial to estimate the user’s gaze [14,17,27,56], similar to the one defined in Equation (1). Such polynomial requires at least nine corresponding pieces of calibration data to solve the 12 unknown coefficients. Nevertheless, there are other inherent polynomial equations used to estimate the user’s gaze. Rattarom et al. [6] and Cerrolaza et al. [16] present two comparative studies which evaluate different polynomial models in terms of accuracy and tolerance to head movements. It is hard to define a general average accuracy of interpolation-based gaze estimation methods because it depends on the code implementation and the eye-tracking data used to evaluate the method. Table A1 shows a summary of the interpolation-based eye-tracking methods used in this paper along with our proposed methods.

**Table A1:** A comparison of traditional interpolation-based gaze estimation methods and proposed compensation methods.

Method	Description	Accuracy	Calibration	Advantages	Disadvantages
Homography	A planar projective mapping between the eye plane and viewed plane	$0.40^{\circ }$–$0.50^{\circ }$	4 targets	It requires only four pieces of calibration data	It is more sensitive to noise, such as camera location
Second-Order Polynomial	A regression which minimizes the sum of squared residuals	$0.50^{\circ }$–$0.60^{\circ }$	9 targets	It is simple to implement and presents good accuracy	It is less accurate than homography-based methods
Camera Compensation	A method to reshape the eye feature distribution in a normalized space	$0.45^{\circ }$–$0.55^{\circ }$	9 targets	It increases the number of high-accuracy gaze estimations	The use of high-order polynomials overfits the model
Distortion Compensation	A method to compensate for the non-coplanarity of $\Pi_{e}$	$0.22^{\circ }$–$0.37^{\circ }$	9 targets	It presents the lowest error in real and simulated scenarios	It can blow up the estimations around the $\Pi_{s}$ boundaries
